# Experimental models of autism spectrum disorders on the example of the use of brain organelles

**DOI:** 10.1192/j.eurpsy.2023.1034

**Published:** 2023-07-19

**Authors:** A. Sidenkova

**Affiliations:** Psychiatry, Ural State Medical University, Yekaterinburg, Russian Federation

## Abstract

**Introduction:**

ASD are heterogeneous pathological conditions characterized by difficulties in establishing social contacts and the manifestation of repetitive behavior. An atypical trajectory of brain maturation, impaired neurogenesis, synaptogenesis, and an imbalance in the excitatory and inhibitory systems of the CNS form the morphofunctional basis of the ASD.

**Objectives:**

To understand the functioning of this complexly organized system in time and space, a three-dimensional model is needed. The closest in vitro model of the human brain from early embryonic stages to aging is brain organoids. Human brain organoids are self-organizing three-dimensional cell aggregates derived from pluripotent stem cells (hiPSCs)

**Methods:**

Organelles generalize neurogenesis, gliogenesis, synaptogenesis, cell migration and cell differentiation, gyrification of the cerebral cortex, and reflect the connections of brain regions.

**Results:**

The use of telencephalon organelles in the RAS model revealed a deficit in neuronal migration, acceleration and disruption of cell cycle synchronization, aberrant cell proliferation, abundant synaptogenesis, temporary deviations in the development of the cortex, increased branching of neurons, unbalanced inhibitory differentiation of neurons, high activity of ion channels is a consequence of a violation of FOXG1 activity. Organelles generalize neurogenesis, gliogenesis, synaptogenesis, cell migration and cell differentiation, gyrification of the cerebral cortex, and reflect the connections of brain regions.The use of telencephalon organelles in the RAS model revealed a deficit in neuronal migration, acceleration and disruption of cell cycle synchronization, aberrant cell proliferation, abundant synaptogenesis, temporary deviations in the development of the cortex, increased branching of neurons, unbalanced inhibitory differentiation of neurons, high activity of ion channels is a consequence of a violation of FOXG1 activity .

**Conclusions:**

hiPSCs can provide insight into the cellular mechanisms underlying ASD as a neuropsychiatric disorder, providing access to the development of platforms for in vitro drug screening and individualized patient therapy.

**Disclosure of Interest:**

None Declared

